# By the Way

**Published:** 1910-07-30

**Authors:** 


### By the Way.

### ORGANOTHERAPY THROUGH THE AGES.

The recent revival of organotherapy in the treatment of disease has led Dr. Gaudichard to publish in the "Répertoire de Pharmacie," an account of some of the compounds of animal organs used in past ages for the same purpose. Dioscorides of Anazarbes, a Greek physician who flourished in the first century, states in his "Materia Medica" that renal disease and dropsy are much benefited by a mixture of honey and dried hedgehog's liver. He also recommends for cough a daily dose of a medicine made up from the lung and palate of a stag dried in dung and crushed with honey to form a lohock, a preparation of a middle consistence between a scft electuary and a syrup, much used at the time on account of its being comparatively pleasant to take. Pliny advised for stomach troubles a paste made from snails, a remedy which still figures in some modern pharmacopœias. The same authority used pig's testicles ground in milk in the treatment of epilepsy. Fox's lung dried and boiled in water yielded a decoction held in much esteem for shortness of breath. Jeoffroy made use of extracts of all kinds of animal organs, which closely resembled those in use at the present time. He administered infusions of liver and heart. For the cure of sterility Joseph du Chesne recommended an extract of ram's testicles made with wine and dried. In his "Elements of Chemistry" Jean Biguin states: "The extracts thus specifically named are derived from animals or vegetables by means of solvents or other appropriate ingredients, such as spirits of wine, water, etc." In cases of incontinence of urine Mésué advised the use of a solution in salt or honey of the urinary bladder. Jean de Cuba gave ox spleen mixed with honey in affections of the spleen. Dusseau, who believed that the brain of birds formed a powerful aphrodisiac, added them to egg-yolk and honey. In the seventeenth century Van Helmont pinned his faith to the virtues of dried blood. In the century following Helvetius prescribed preparations of animal organs in the form of soups, macerations and extracts. Later Baudron in his Pharmacopœia says, "The lohock of lung is prepared by mixing with sugar equal quantities of fox's lung, liquorice juice, maidenhair, and the seeds of fennel and anise." It will thus be seen that our professional forbears, acting purely empirically, anticipated many of the animal extracts now used in organotherapy.

### VAMPIRES.

Before being stationed in French Guiana Dr. A. Guillon, a surgeon-major in the French colonial medical service, shared the opinion commonly held by educated Europeans, that the tales told of vampires rested for the most part on legendary foundations. In an interesting article which he contributes to a recent number of "La Clinique" he states the reasons which have led him to reconsider this opinion. The fact that he was called upon almost from the moment of his arrival to treat wounds said to have been caused by the bites of these animals did not at first induce him to believe in the truth of their supposed cause, since the victims could never produce the author of the mischief, nor were they able to assert that they had seen the bats bite them. When, however, he had been sufficiently long in the place to see the large number of wounds in both men and animals attributed to the bite of the vampire his opinion gradually veered round, and he began to look out for their causal agent. This he concluded to be a bat, the Phyllostoma vampirus, an insectivorous mammal of the order Cheiroptera, of the Phyllostomian tribe and the vampire family. He had some considerable difficulty in recognising the animal owing to the lack of an adequate reference library. This bat possesses a nasal appendix oval in shape and hollowed out into a funnel, which gives it a peculiarly diabolical appearance, well brought out in the drawings reproduced. It is universally feared both by Europeans and natives in the colony, and is particularly hurtful to small animals, poultry and game, though also attacking horses and cattle. Its depredations are carried out at night and in the dark, the presence of lamp-light being sufficient to drive the animal away, for which reason poultry-houses and cattle-sheds in the district are always provided with lamps at night.—The wounds it causes are found in poultry on the back and legs, in horses on the head and neck and in front of the withers, and in human beings on the legs, feet, and especially the toes. The bites cause little, if any, pain, and the wound is small, though it bleeds severely, suggesting that the animal secretes some anti-coagulation body similar to that of leeches. The victim does not usually wake at the time the wound is caused, but only discovers its presence in the morning. The bite is not dangerous to adult human beings and cattle, though repeated bleedings at short intervals of time may render children anæmic, and two consecutive bites have been known to kill chickens from loss of blood. The chief dangers lie in sepsis and the opening of a large vein. The author suggests that the occurrence of this last accident may explain some of the legends of death from a vampire's bite. He has not been able to obtain personally any direct evidence that the bat is responsible for the wounds, but on one occasion a sister attached to a local hospital, and whose evidence was in all respects trustworthy, reported that on going into the hen-house one morning at 4 a.m. she removed a vampire from the leg of one of the hens. Post-mortem examination of the bat never revealed the presence of undigested blood in the stomach or intestines, nor could a vampire be induced to bite a guinea-pig confined with it in a cage. The author is nevertheless convinced that the animal is the real cause of the bites shown to him by Europeans and natives, and seen by him on animals. Prophylaxis consists in the use of strong mosquito-netting properly secured all round the bed, and in animals the use of lighted lamps in their stables. For the wounds the usual antiseptic methods are all the treatment required. Diagnosis is rarely called for except in the case of new arrivals, the patient usually supplying it himself. Prognosis, apart from the risk of sepsis, is good.

### WHY SOAP CLEANSES.

Some interesting details are given in *La Technique Moderne* concerning the researches of M. W. Spring, a Belgian chemist, into the processes whereby soap cleanses. It has been stated that the cleansing properties of soap are due to its combination with the soiling substance, but this only half explains the question as to how soap acts. An explanation is still required as to why it disappears after having taken up and entered into combination with the dirt, or, in other words, why the compound is removed. The author, after studying the action of soap upon various soiling substances, such as lampblack, clay, red chalk, silica, cellulose, etc., comes to the conclusion that the cleansing of an object consists in a process of substitution. There is brought about a colloidal combination of the soap and the soiling substance, which, by reason of its constitution, no longer has the power of fixing itself by absorption on to the solid body, with the result that it is easily carried away by the water. Thus in washing with soap one puts it in contact with one's soiling substances, these latter having a greater affinity for the former than for one's skin. The process does not end here, for soap in its turn has even a greater affinity for one's skin than for the substances, which it proceeds to replace and to set free to be taken up by the water and removed. The dirty substances have a chemical action on the fresh solution of soap and water, resulting in the formation of an acid salt, with which they agglutinate themselves. This compound of colloidal absorption is due to electrical action. The constituents have different electrical polarities. The compound is stable and can pass through a filter-paper without soiling it.

